# Rare Monteggia Type IV Fracture With Posterior Radial Head Dislocation and Concomitant Glenoid Fracture: A Case Report

**DOI:** 10.7759/cureus.83893

**Published:** 2025-05-11

**Authors:** Taha El Aissaoui, Achraf Tebbaa El Hassali, Adnane Lachkar, Najib Abdeljaouad, Hicham Yacoubi

**Affiliations:** 1 Orthopedics and Traumatology, Mohammed VI University Hospital, Faculty of Medicine and Pharmacy, Mohammed First University, Oujda, MAR

**Keywords:** bado classification, forearm double fracture, monteggia, radial head dislocation, radial head resection

## Abstract

Monteggia fractures are uncommon ulnar fractures with associated radial head dislocation, which are classified into four Bado types. Type IV lesions, involving both forearm fractures with anterior radial head dislocation, are particularly rare. This case describes an exceptionally rare variant in a 23-year-old male patient following a motor vehicle accident: a Bado Type IV Monteggia fracture with posterior radial head dislocation, accompanied by an ipsilateral Ideberg type III glenoid fracture, which was initially missed. Despite prior open reduction and internal fixation (ORIF) of the forearm shafts and non-operative glenoid management, the patient presented to our department with significant elbow and shoulder stiffness and a persistent posterior radial head dislocation. Salvage radial head resection via a lateral Kocher approach, followed by intensive rehabilitation, yielded significant functional recovery at six months, including full elbow range of motion (except for 40° pronation) and marked improvement in shoulder mobility. The patient remained pain-free. This case underscores the diagnostic challenges of atypical Monteggia fracture patterns in complex trauma and supports radial head resection as a viable salvage option in chronic, symptomatic dislocations, even in this unique variant, thus expanding the current understanding of the spectrum of Monteggia injuries.

## Introduction

A Monteggia fracture is defined as a proximal ulna fracture associated with a radial head dislocation [1,2]. Bado classified these injuries into four types based on the direction of the radial head dislocation and the angulation of the ulnar fracture [1]. These injuries are frequently underdiagnosed. According to one study, approximately 25.5% of cases were initially missed by radiology teams; 14.9% by bedside clinicians, including orthopedic surgeons; and 10.6% by both groups [3]. Delayed or missed diagnosis may result in significant complications, most notably persistent radial head dislocation [3]. Posterior radial head dislocation is most commonly associated with Bado type II injuries. However, the combination of a Monteggia type IV fracture pattern with posterior radial head dislocation has not been well documented in the literature. We present a rare case of such a variant in an adult patient with an initially missed diagnosis, emphasizing the diagnostic challenges and exploring the implications for clinical management.

## Case presentation

We report the case of a 23-year-old male patient without any significant prior medical history who sustained a complex left upper limb injury following a motor vehicle accident. The injury included an ipsilateral Ideberg type III glenoid fracture and a Monteggia type IV fracture associated with posterior radial head dislocation. Initial surgical management consisted of open reduction and internal fixation (ORIF) of the ulnar and radial shafts using plates. The glenoid fracture was managed non-operatively with immobilization. These procedures were performed three months before the patient's presentation at our department.

At the time of consultation at our department, the patient reported no improvement in elbow range of motion and presented with complete stiffness of the ipsilateral shoulder.

**Figure 1:**
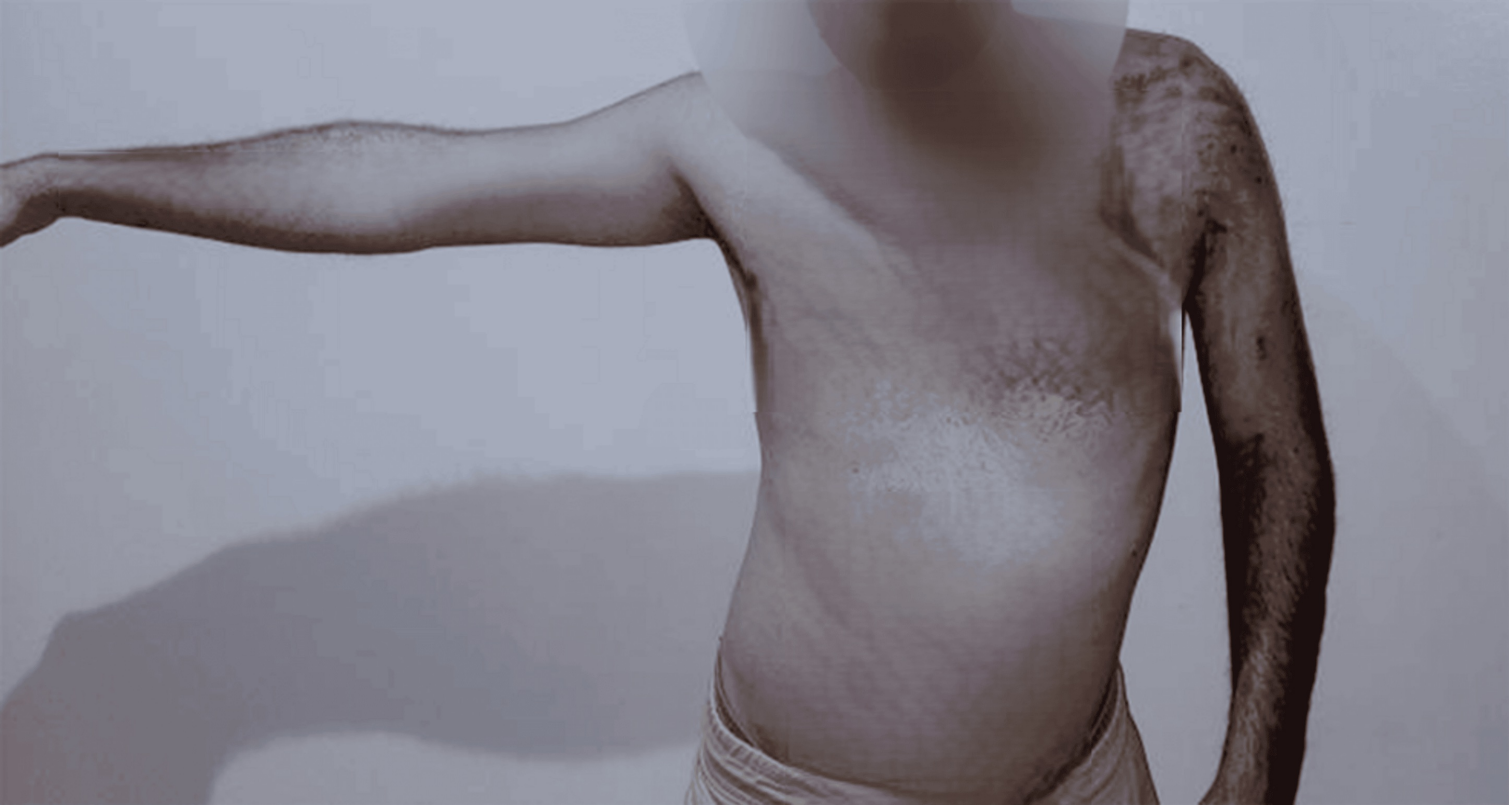
Clinical image showing limited left shoulder abduction

**Figure 2:**
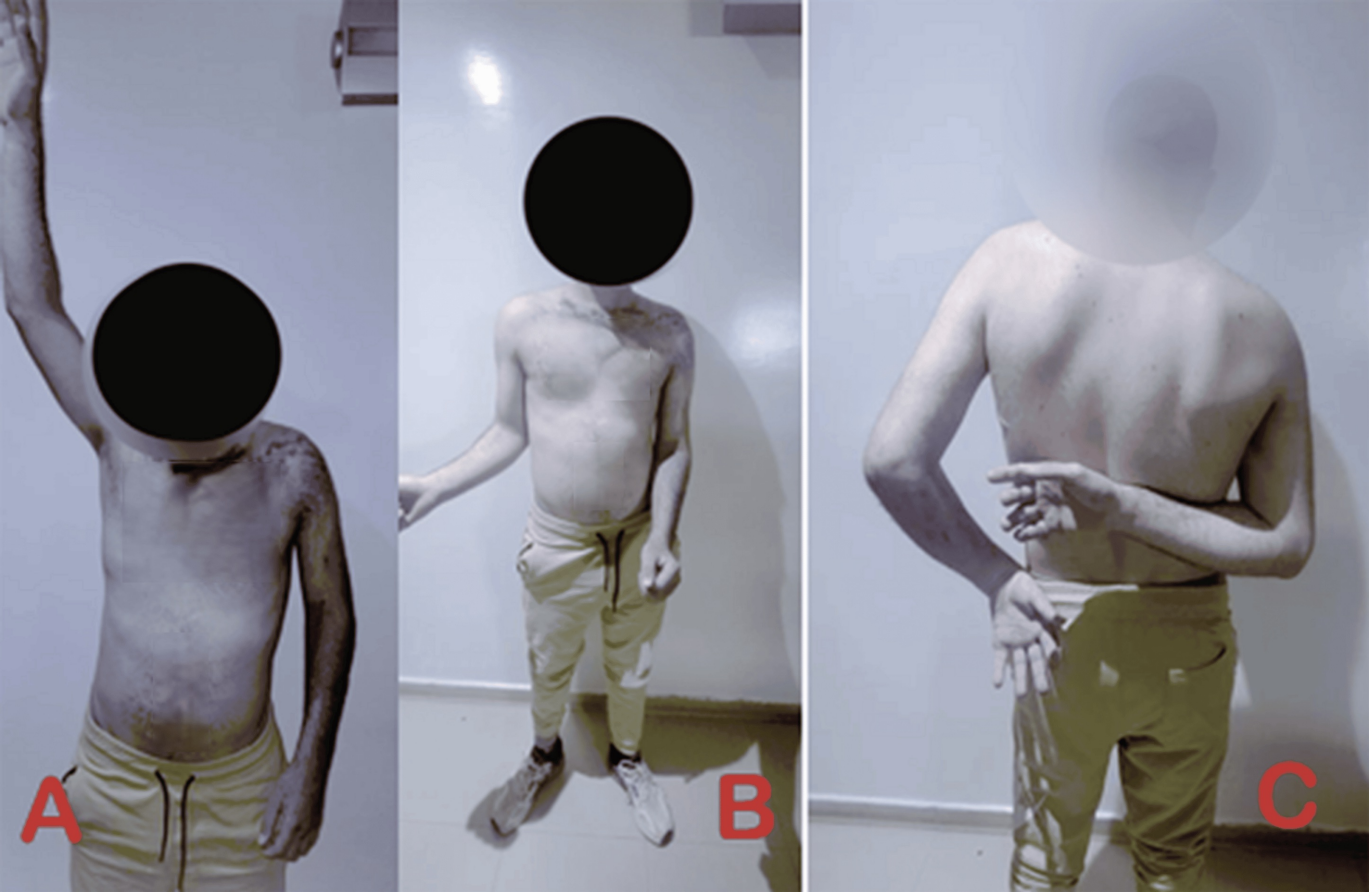
Clinical image showing a limited active range of motion of the left shoulder (A) Limited left shoulder flexion. (B) Limited left shoulder external rotation. (C) Limited left shoulder internal rotation.

**Figure 3:**
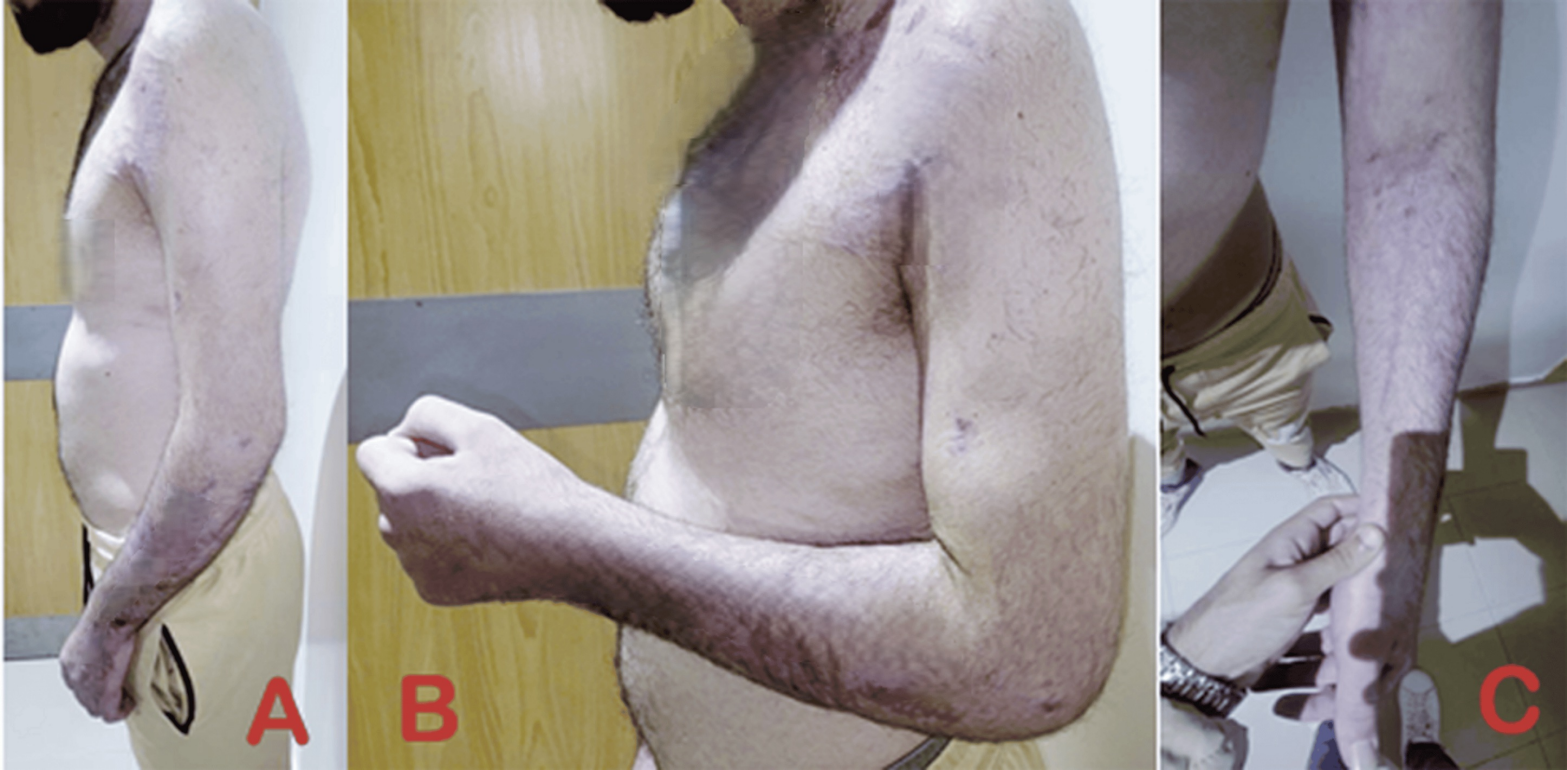
Images showing a limited active range of motion of the left elbow (A) Restricted extension of the left elbow. (B) Restricted flexion of the left elbow. (C) Restricted pronosupination of the left elbow.

Physical examination revealed multiple scars over the shoulder and forearm, including two distinct incisions over the forearm. Significant atrophy was noted in the supraspinatus and infraspinatus fossae. The patient demonstrated complete loss of active shoulder motion and restricted elbow motion, with an extension deficit of 30°, flexion limited to 90°, and complete loss of supination. Vascular and neurological assessment of the left upper limb was unremarkable (Figures 1-3).

**Figure 4:**
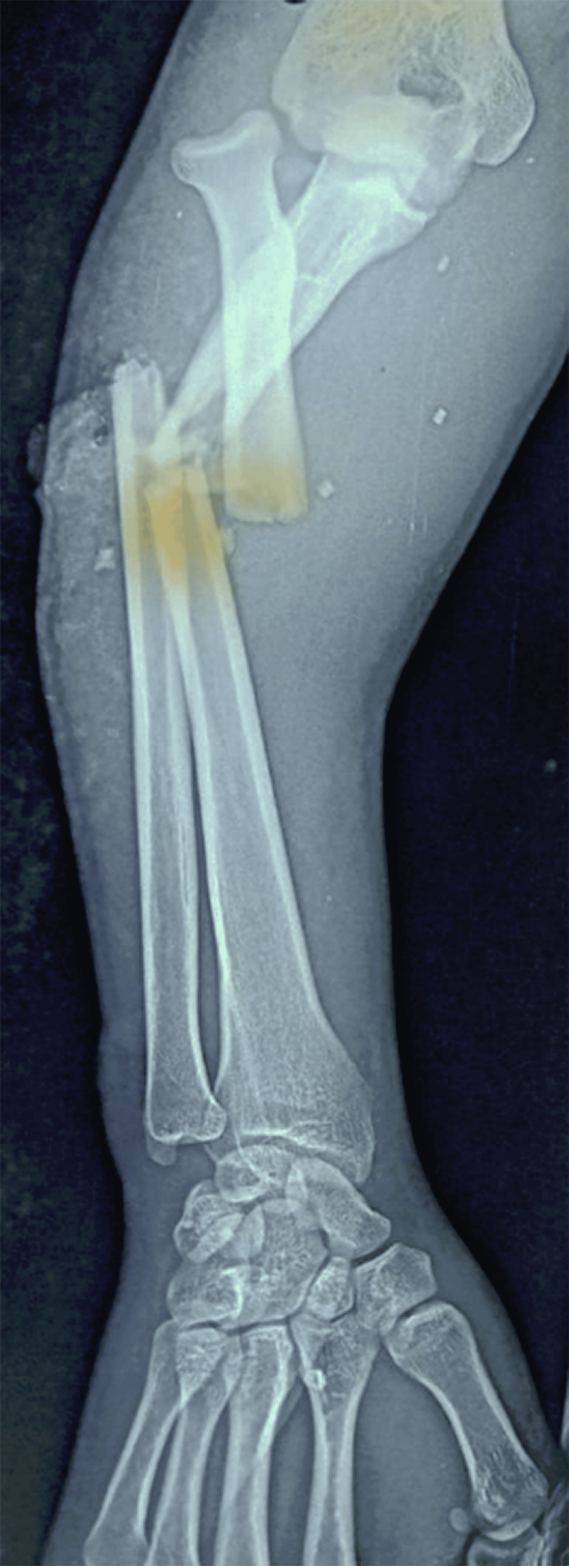
Anteroposterior view of the left forearm showing a type IV Monteggia fracture

**Figure 5:**
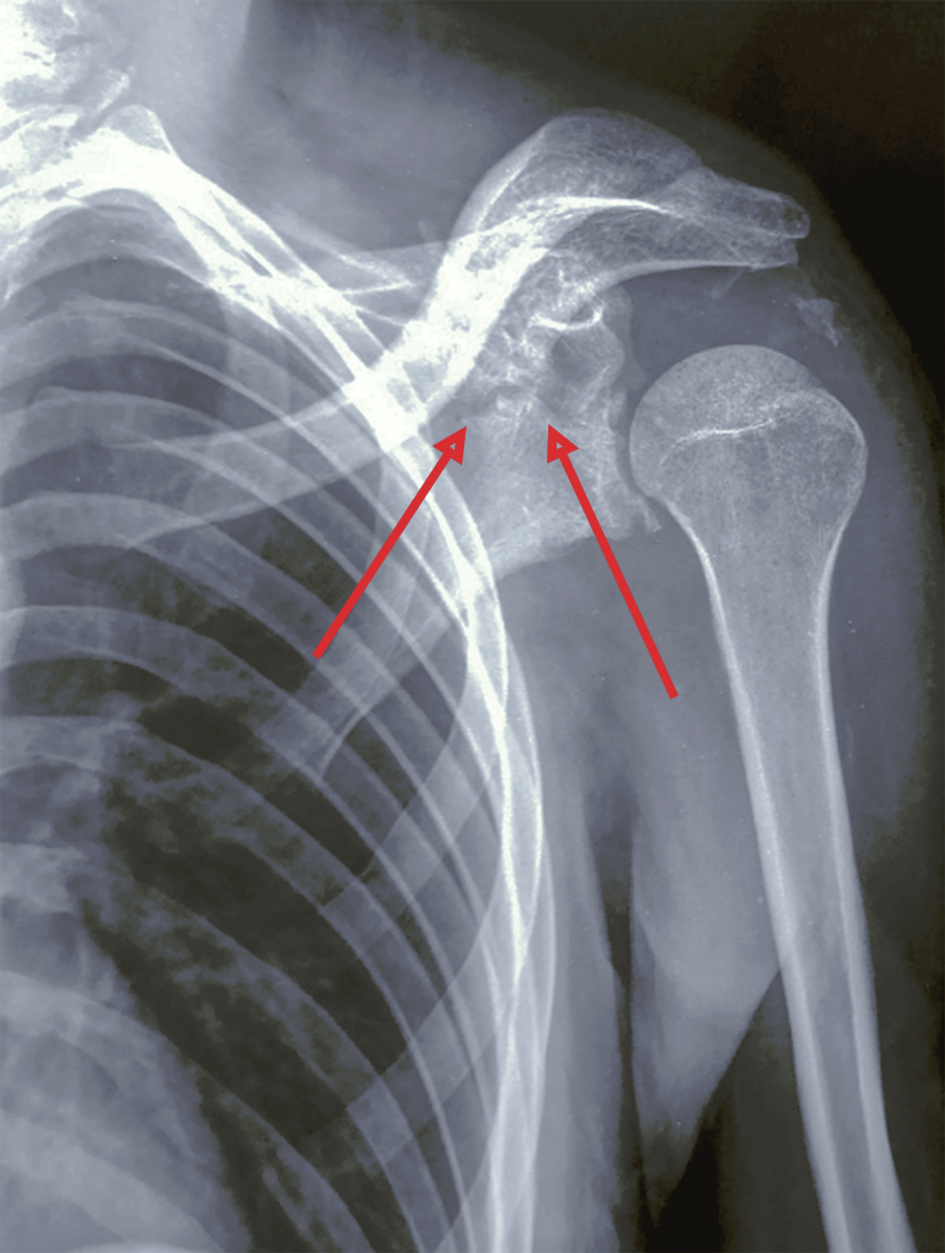
Anteroposterior radiograph of the left shoulder showing an Ideberg type III glenoid fracture

**Figure 6:**
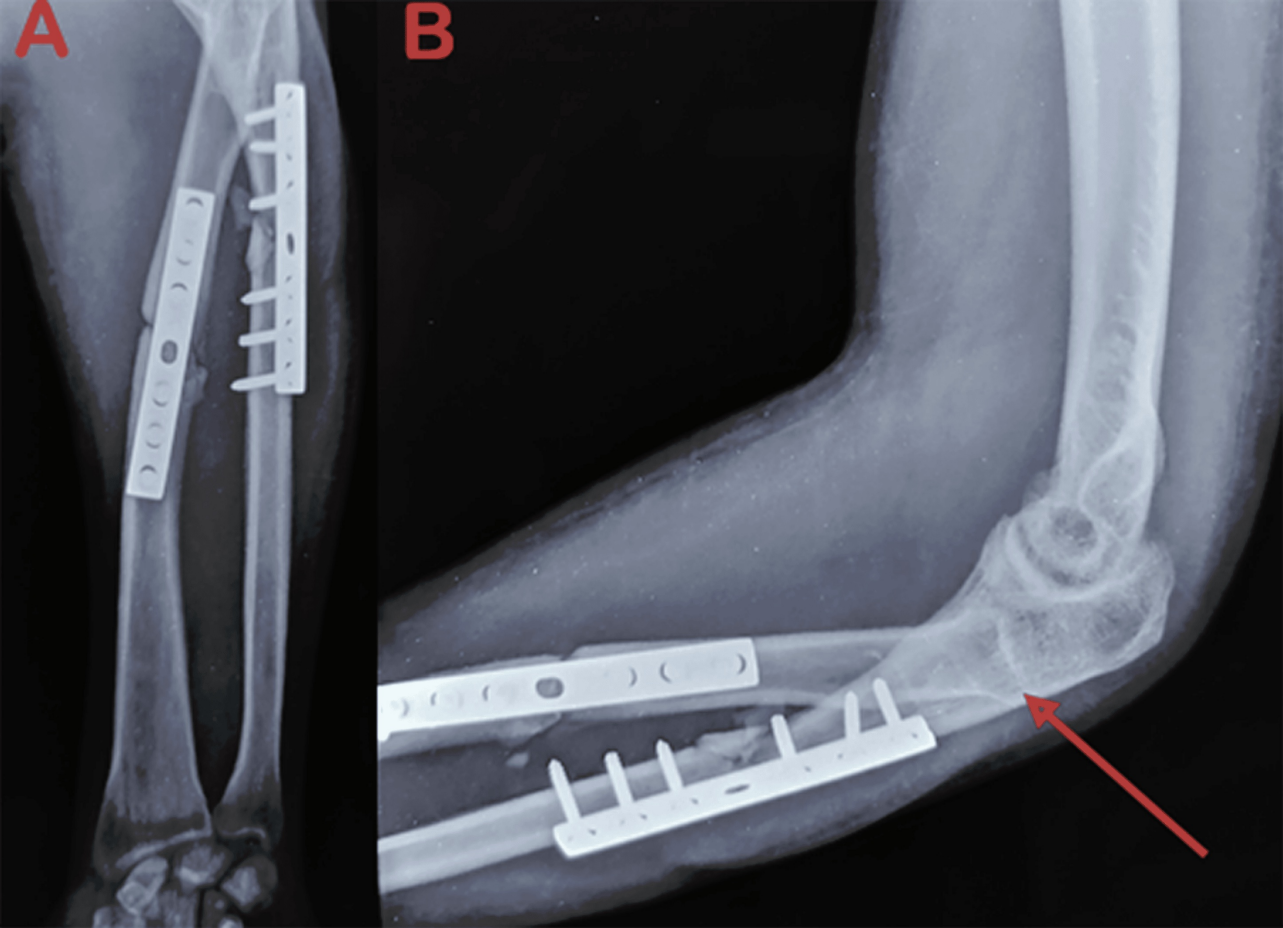
Radiographic view of the left elbow and forearm (A) Anteroposterior view of the left forearm showing a well-aligned ulnar and radial shaft. (B) Lateral view of the elbow and forearm showing a posterior dislocation of the radial head.

Initial radiographs taken at the time of trauma confirmed the diagnosis of a Monteggia type IV fracture and an Ideberg type III glenoid fracture (Figures 4, 5). Radiographs obtained at our institution revealed a persistent posterior dislocation of the radial head despite appropriate alignment and fixation of the radial and ulnar shafts with plates (Figure 6).

**Figure 7:**
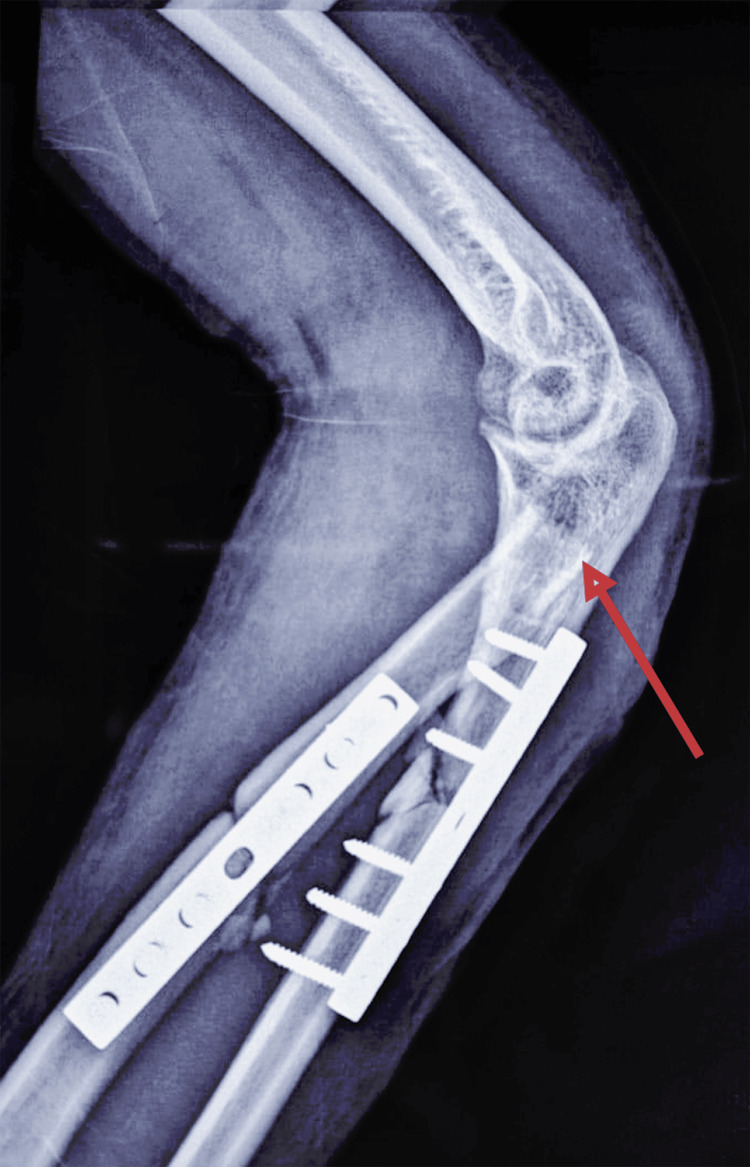
Postoperative lateral radiograph of the left elbow showing radial head resection

Given the delayed presentation of the radial head dislocation, the chronicity of the lesion, the appropriate alignment of the radius and ulna, and the stability of the medial column of the elbow, radial head resection was indicated. The procedure was performed via a lateral Kocher approach, followed by intraoperative elbow mobilization under general anesthesia (Figure 7).

Postoperatively, the patient was enrolled in an early intensive rehabilitation program, which included active and passive exercises to restore the range of motion in the shoulder and elbow, enhance muscle strength, and facilitate recovery through physiotherapy.

**Figure 8:**
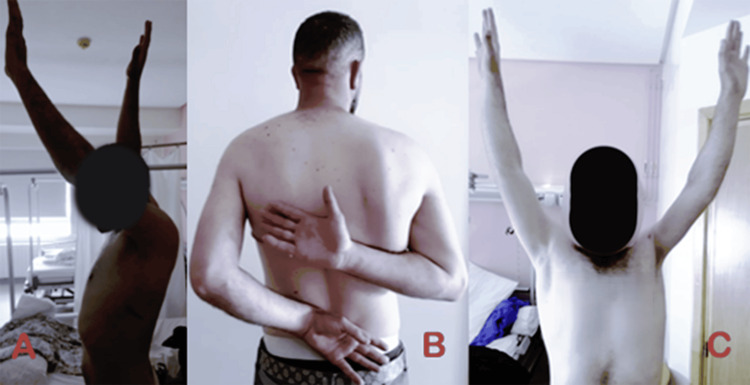
Clinical images demonstrating improved active range of motion of the left shoulder (A) Improved left shoulder flexion. (B) Improved left shoulder internal rotation. (C) Improved left shoulder abduction.

**Figure 9:**
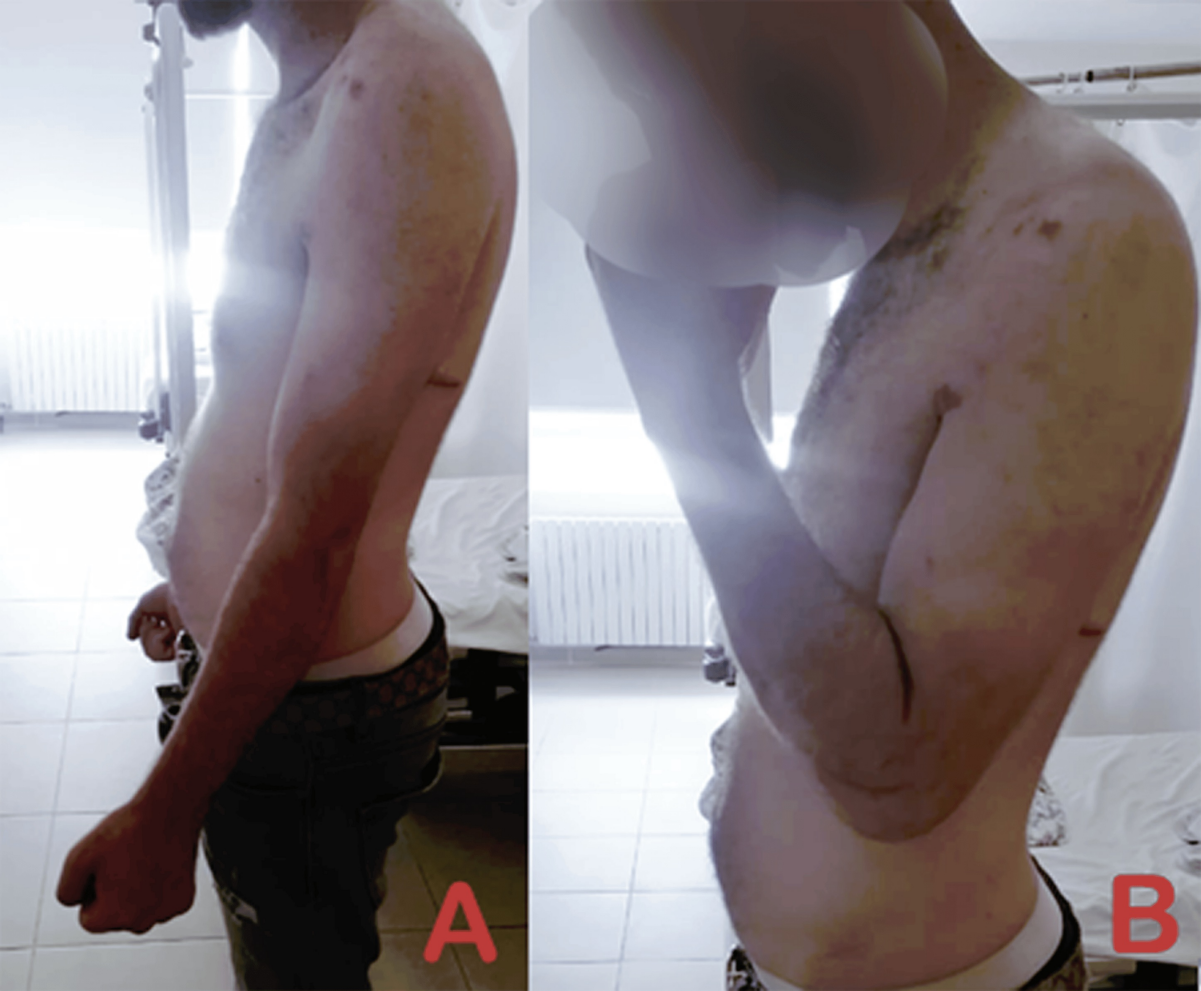
Clinical image showing improved range of motion of the left elbow (A) Improved left elbow extension. (B) Improved left elbow flexion.

**Figure 10:**
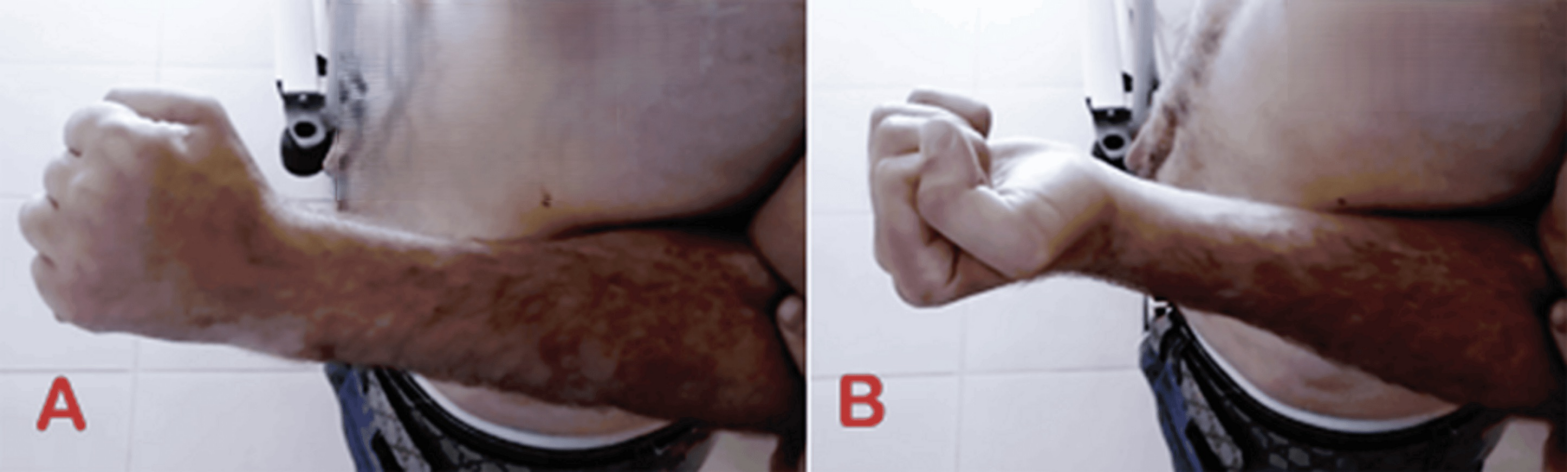
Clinical image showing improved range of motion of the left elbow (A) Improved left elbow pronation. (B) Improved left elbow supination.

**Figure 11:**
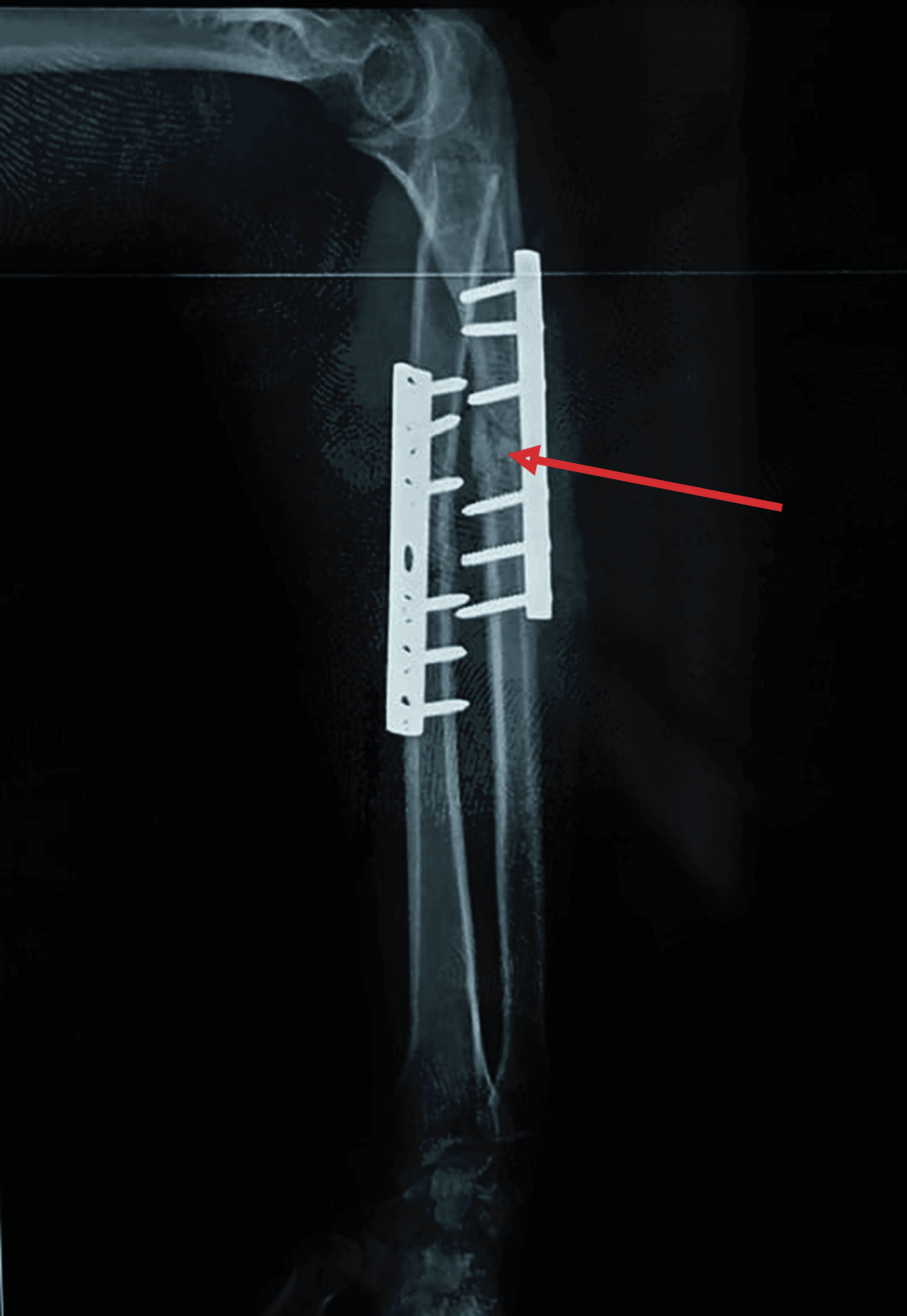
Left forearm radiograph showing evidence of fracture union

After a six-month follow-up, the patient exhibited significant functional recovery. Elbow function included full flexion and extension, 65° supination, and 40° of pronation. Shoulder mobility included 120° of flexion, 150° of abduction, internal rotation sufficient to place the hand behind the back, and 20° of external rotation (Figures 8-10). These functional improvements were accompanied by favorable radiographic findings (Figure 11). The patient remained pain-free and reported no postoperative complications.

## Discussion

Monteggia fractures represent a relatively uncommon injury, accounting for 2%-5% of all proximal forearm fractures [4]. Among these, Bado type IV lesions, characterized by concurrent fractures of both the radius and ulna with radial head dislocation, are particularly rare, constituting approximately 1% of all Monteggia fractures [5]. Typically, radial head dislocation in type IV injuries occurs anteriorly. However, the present case exhibits an exceptional posterior dislocation of the radial head, a pattern more frequently associated with Bado type II injuries. To the authors' knowledge, the combination of a Monteggia type IV fracture with posterior radial head dislocation has not been previously documented in Morocco and represents the second case reported following the one described by Bhandari and Jindal [6], underscoring the unique nature of this presentation.

Despite the documented rarity of type IV lesions, clinicians should conduct a thorough evaluation of the elbow joint in all cases of midshaft forearm fractures to avoid overlooking this complex injury.

Radial head resection is generally indicated in cases of irreparable radial head fractures or chronic dislocations where reconstructive procedures are not feasible [7]. While potential complications such as elbow instability, proximal radial migration, and degenerative changes have been noted, several studies have reported favorable outcomes following radial head excision in carefully selected patients. For example, a series analyzing Monteggia lesions in adults reported significant functional improvement in specific cases of chronic dislocation treated with radial head excision [8].

The patient demonstrated significant functional recovery following radial head excision and a structured rehabilitation program in the present case. At the six-month postoperative assessment, the patient exhibited full elbow flexion and extension, complete supination, and 40° of pronation. Furthermore, marked improvements were observed in shoulder mobility, with 120° of flexion, 150° of abduction, internal rotation allowing the hand to be placed behind the back, and 20° of external rotation. The patient remained pain-free and reported no postoperative complications.

This case underscores the critical importance of meticulous evaluation and timely management of atypical Monteggia fracture patterns. Furthermore, it suggests that radial head excision can be a viable treatment strategy in selected chronic radial head dislocation cases where reconstruction is not feasible, potentially leading to satisfactory functional outcomes.

## Conclusions

This case report highlights a rare and previously underdocumented variant of a Monteggia type IV fracture characterized by a posterior radial head dislocation associated with a glenoid fracture. The successful outcome following delayed radial head resection and rehabilitation underscores the potential viability of this approach in select cases of chronic, symptomatic radial head dislocation, even in the context of an atypical Monteggia injury. However, the initial missed diagnosis emphasizes the diagnostic challenges associated with complex upper extremity trauma and the importance of a thorough radiographic evaluation of the entire forearm and elbow joint. Further research and reporting of similar cases are warranted to better understand the biomechanics, optimal management strategies, and long-term outcomes of this unique injury pattern.
